# Politics of Envy? Meritocracy Beliefs, Not Envy, Drive Support for Redistribution

**DOI:** 10.1177/01461672251410720

**Published:** 2026-01-08

**Authors:** Jasper Neerdaels, Lisa Blatz, Jan Crusius

**Affiliations:** 1 University of Greifswald, Germany; 2 KU Leuven, Belgium

**Keywords:** Envy, ideology, redistribution, system justification, inequality

## Abstract

Support for redistribution is often dismissed as driven by a morally questionable motive: Malicious envy. Seemingly supporting this notion, in some studies, liberalism was correlated with envy, and envy predicted support for redistribution. However, we argue that these results can be explained by meritocracy beliefs rather than envy; specifically, we hypothesize that liberals are only indirectly prone to envy to the extent that they believe wealth is often not merited. Consequently, we argue that these meritocracy beliefs drive redistribution support, not envy. We found support for our predictions in three surveys and one experiment (total *N* = 4,171), showing that (a) liberalism only indirectly predicted envy via lowered meritocracy beliefs, and (b) meritocracy beliefs, not envy, (negatively) predicted support for redistribution. Moreover, when an experimental manipulation increased liberals’ perceptions of wealth as deserved, their support for redistribution decreased. These findings may inform a more evidence-based debate amid growing inequality.

## Introduction

Economic inequality has wide social and health costs (Jetten et al., 2021; Wilkinson & Pickett, 2017), yet public support for redistribution is often relatively low (García‑Sánchez et al., 2020; Macdonald, 2020; Witko & Moldogaziev, 2025). Critique often centers on perceived coercion, potential adverse economic effects, or distrust of the government (Alesina & Giuliano, 2011; Alesina & Glaeser, 2010; Kirchler, 1999; Witko & Moldogaziev, 2025). Another widespread argument, especially popular in political commentary, claims that redistribution is driven by envy, an aversive response to unfavorable comparison (Harris & Henniger, 2013; Schoeck, 1987). Perhaps most famously, Winston Churchill (1950, p. 347) called socialism “the gospel of envy.”

The argument is made on two grounds: some say that liberals (i.e., left-wing individuals), who are traditionally in favor of redistribution, are especially prone to envy (Harris & Henniger, 2013; Hoogland, 2016); others argue that envy is one of the reasons why individuals support redistribution (Kemp & Bolle, 2013; Lin & Bates, 2021, 2024; Sznycer et al., 2017). The “politics of envy” argument is widespread. For example, Harris and Henniger (2013) reported that 621 U.S. newspaper articles mentioned the phrase “politics of envy” in the 5 years before their publication. However, despite the popularity of the narrative, empirical research is surprisingly inconclusive, and empirical links between liberalism and envy, and between envy and redistribution, are mixed (Harris & Henniger, 2013; Lin & Bates, 2021). Accordingly, the present research seeks to clarify the relationship between liberalism, envy, and support for redistribution. In doing so, we argue that a central mechanism has been overlooked in previous studies and discussions: meritocracy beliefs concerning the deservingness of wealth. We posit that this mechanism can explain why previous results were inconclusive; specifically, we propose that liberalism and envy are only indirectly linked. More specifically, we hypothesize that liberals are less likely to subscribe to meritocracy beliefs and thus more likely to perceive wealth as undeserved. Such beliefs are also correlated with envy, while liberalism per se, we argue, is not. Critically, we posit that it is perceived unfairness, particularly the perception that wealth is not based on merit, that drives support for redistribution, not envy. Malicious envy is, in fact, often accompanied by the sense that the advantaged do not deserve their status, but, importantly, it is not reducible to unfairness perceptions (Smith & Kim, 2007). However, because of mere conceptual overlap among measures, earlier links between envy and redistribution may reflect perceived lack of merit rather than envy per se (Lange & Crusius, 2015b; Van de Ven et al., 2012).

We test this account across two preregistered U.S. surveys, one preregistered experiment, and a large German survey. Our findings contribute in two ways: (a) They show that liberals are merely indirectly prone to envy because they are less likely to endorse meritocracy beliefs; and (b) they clarify that meritocracy beliefs, rather than envy, explain support for redistribution. More broadly, these findings may foster a more nuanced, evidence-based discussion of motives underlying redistribution debates and help inform policymaking around inequality.

## Liberalism and Envy

Consistent with usage in U.S. contexts and prior work on the “politics of envy” (Archer et al., 2022; Duckitt & Sibley, 2009; Graham et al., 2009; Harris & Henniger, 2013; Jost et al., 2003), we use “liberal” to refer to left-leaning and “conservative” to right-leaning orientations. In the standard left–right framework, conservatives tend to value stability and tradition and are more accepting of economic inequality, whereas liberals are more open to change and emphasize social equality and justice (Jost et al., 2003; Kteily & Brandt, 2024).

Critics, often from conservative circles, dispute such benevolent interpretations of liberal motivations. Rather than viewing liberals’ support for redistribution as rooted in principled concerns about fairness, they argue that it reflects envy of the wealthy, that is, a desire to level incomes not for reasons of justice but out of malicious intentions (Ahier & Beck, 2003; Harris & Henniger, 2013; Nielsen & Axelsen, 2022). This allegation forms the core of the “politics of envy” narrative.

Envy is a negative emotional response to another’s superiority in possessions, achievements, or qualities, characterized by wanting their advantage or wishing they lacked it (Lange & Crusius, 2015a; Smith & Kim, 2007; Smith et al., 1999). As one of the seven deadly sins, it is widely viewed as immoral (Ahier & Beck, 2003; Lange & Crusius, 2015b; Lange & Protasi, 2024; Thomason, 2015). Contemporary work distinguishes malicious from benign envy. Malicious envy corresponds to traditional accounts: it involves painful inferiority, perceptions of undeserved advantage, and hostile wishes that the superior lose their position (Crusius & Lange, 2021; Lange, Weidman, et al., 2018). Benign envy is comparably painful but motivates self-improvement rather than harm (Crusius & Lange, 2021; Lange, Weidman, et al., 2018). Most discussions linking envy to liberalism or redistribution focus, explicitly or implicitly, on malicious envy (Harris & Henniger, 2013; Hoogland, 2016; Lin & Bates, 2021; Sznycer et al., 2017). Accordingly, we focus here on “envy proper” (Smith & Kim, 2007), that is, malicious envy.

Despite its popularity, the claim that liberals are more envious lacks strong empirical support. Harris and Henniger (2013) found a small correlation between liberalism and dispositional envy, which disappeared after controlling for age. They concluded that older individuals tend to be more conservative and less envious, suggesting that age explained the correlation. Hoogland (2016) found similarly mixed results: some studies linked liberalism to malicious envy, while others did not. In some cases, conservatives even expressed *more* envy, specifically when the target had inherited (rather than earned) their wealth (Hoogland, 2016).

## Envy and Support for Redistribution

Beyond claims that liberals are especially prone to envy, the “politics of envy” argument also holds that envy motivates support for left-wing policies, especially redistribution (Protasi, 2021). Redistribution aims to transfer resources from the wealthy to the poor through policies such as taxation, welfare, or insurance (Durante et al., 2014; García-Sánchez et al., 2020).

However, empirical tests of whether envy predicts support for redistribution yielded mixed results. Kemp and Bolle (2013) found small, inconsistent effects in samples from Germany and New Zealand, and Sznycer et al. (2017) reported small correlations in three of four studies. These studies used the Dispositional Envy Scale (DES; Smith et al., 1999), which treats envy as a unidimensional blend of painful inferiority and resentment.

In a replication of Sznycer et al. (2017), Lin and Bates (2021) found no significant association between the DES and support for redistribution. They argued that the DES may not sufficiently capture ill-will components of envy; therefore, *malicious* envy may be a more relevant construct (Lin & Bates, 2021; see also Archer et al., 2022; Hoogland, 2016). Measuring malicious envy (Lange & Crusius, 2015a, 2024), Lin and Bates (2021) indeed observed small, significant effects on redistribution, a pattern they replicated in two later studies (Lin & Bates, 2024).

In summary, although the “politics of envy” narrative is widespread, empirical support remains limited. Consequently, this article aims to clarify the relation between liberalism, envy, and support for redistribution.

## “Politics of Envy”: The Role of Meritocracy Beliefs

Building on the findings outlined above, we argue that to understand the relationship between envy and liberalism, it is important to consider a psychological mechanism that is strongly related to political ideology and support for redistribution, but also envy: meritocracy beliefs. Specifically, we argue that liberals are more likely to perceive wealth as based on undeserved advantages rather than merit. This belief, not liberalism per se, is related to malicious envy. More importantly, we argue that these deservingness beliefs primarily drive and thus predict support for redistribution, not envy.

Our argument draws on extensive research on system justification beliefs, that is, the view that social structures and hierarchies are fair, that discrimination is minimal and opportunities equal, and that people generally receive what they deserve (Jost, 2019; Napier & Jost, 2008). Such beliefs can serve adaptive functions by reducing uncertainty, deflecting personal responsibility for negative outcomes, and enhancing subjective well-being (Napier & Jost, 2008; Neerdaels et al., 2024). Meritocratic ideology and its belief system can be seen as a relatively stable predisposition, providing a lens through which individuals make judgments, for example, regarding the deservingness of wealth, in specific situations in everyday life. Accordingly, a core component of system justification, and meritocratic ideologies in general, is the belief that hard work pays off, rendering wealth differences justifiable as reflections of individual effort (Jost, 2020). Such beliefs are embedded in ideological systems like the Protestant work ethic (PWE), which asserts that people can “pull themselves up by their bootstraps” through hard work (Jost, 2020; Levy et al., 2006; McHoskey, 1994). The term PWE was coined by Weber (2002) to describe the change of attitudes toward work and its rewards that Protestantism evoked in the world, but has since developed into a general system justification framework. Today, PWE is prevalent not only among Protestants but also across many societies, including the United States, Europe, and also some Muslim-majority countries (Hayward & Kemmelmeier, 2011; Shirokanova, 2015).

A wealth of studies indicates a strong relationship between conservatism and meritocracy beliefs (Furnham & Bland, 1983; Jost, 2019, 2020; Jost et al., 2003; Kteily & Brandt, 2024; Napier & Jost, 2008). For example, conservatives value stability and tradition, which makes them more likely to believe that the system is legitimate and fair and therefore worth preserving. Conservatives also tend to believe that social hierarchies are acceptable and natural. Consequentially, they are likely to subscribe to meritocracy beliefs which indicate that social inequality is justified because it reflects differences in individual effort and talent, and is even desirable because it can motivate people to do their best to succeed (Furnham & Bland, 1983; Jost, 2019; Jost et al., 2003; Kteily & Brandt, 2024; Napier & Jost, 2008). Liberals, in contrast, place special emphasis on equality and fairness and believe that society has an obligation to take care of disadvantaged groups (Graham et al., 2009). Accordingly, liberals are more sensitive to *perceived injustices* than conservatives. For example, liberals are more likely to view wealth and power as resulting from undeserved privilege via, for example, gender, ethnic group, or luck (Gromet et al., 2015; Jost, 2019; Jost et al., 2008). Thus, liberals tend to believe that wealth is likely *not* mainly a deserved result of hard work (Gromet et al., 2015; Napier & Jost, 2008). It is these beliefs, we argue, that indirectly relate liberalism to malicious envy: Studies show that malicious envy is likely to occur when individuals are confronted with a superior who is perceived not to deserve their advantage (Lange & Crusius, 2015b; Lange, Weidman, et al., 2018; Van de Ven et al., 2012). In such a situation, people are likely to feel that it is morally legitimate for the other person to lose their unfair advantage. Thus, liberals’ tendency to question the deservingness of wealth should be indirectly linked to proneness toward experiencing malicious envy. Importantly, given the weakness of the empirical evidence regarding a relationship between liberalism and envy, we argue that, if at all, this effect should be largely indirect; thus, liberals should only be more likely to experience envy *inasmuch* as they believe that wealth is undeserved. In contrast, the “politics of envy” argument would imply that liberalism should predict envy above and beyond deservingness perceptions.

### Meritocracy Beliefs, Envy, and Support for Redistribution: The Present Study

Turning to the second component of the “politics of envy” argument, we propose that (low) meritocracy beliefs, not envy, drive support for redistribution. We base this claim on both empirical and theoretical grounds. Empirically, as noted above, studies show that mainly *malicious* envy predicts support for redistribution (Lin & Bates, 2021, 2024). A key distinction of malicious envy is the appraisal that the other’s advantage is undeserved (Lange, Weidman, et al., 2018; Van De Ven et al., 2012). While meritocracy beliefs and malicious envy are distinct constructs, they overlap significantly in that both involve judgments about deservingness. A range of studies show that meritocratic ideology, and specifically beliefs about the deservingness of wealth, are related to support for redistribution (Alesina & Glaeser, 2010; Mengel & Weidenholzer, 2023). People are more supportive of redistribution when they believe that the wealthy benefit from privilege rather than hard work, and the poor are not primarily responsible for their disadvantage.

Accordingly, we argue that the observed effect of malicious envy on support for redistribution may, in fact, be driven by underlying (low) meritocracy beliefs. However, there are also theoretical reasons to question the “politics of envy” argument. On one hand, studies show that liberals tend to be more prosocial and empathetic than conservatives, which contradicts a tendency toward malicious envy (Hasson et al., 2018; Van Lange et al., 2012). On the other hand, if redistribution supporters were driven by envy, they would likely be of lower socioeconomic status, since envy involves upward comparison, or be more materialistic, neither of which is supported by evidence (Andersen et al., 2023; Hoy & Mager, 2021; Kim et al., 2018). Instead, we argue that support for redistribution stems from the belief that wealth is not primarily deserved: if the wealthy are seen as advantaged due to favors, privilege, or luck rather than hard work, then redistribution is likely viewed as just. If one has benefited from societal structures, it may seem only fair that they give back to society and share their wealth with those who are less fortunate.

**Hypothesis 1:** Liberalism is indirectly related to malicious envy via (low) meritocracy beliefs.
**Hypothesis 2:** The effect of malicious envy on support for redistribution is smaller than the (negative) effect of meritocracy beliefs (on support for redistribution).
**Hypothesis 3:** In a hypothetical meritocratic world (vs. a control condition), the relationship between liberalism and support for redistribution decreases.
**Hypothesis 4:** In a hypothetical meritocratic world (vs. a control condition), liberals’ tendency to view wealth as undeserved is reduced and, consequently, so is their support for redistribution.

## Methods

We tested our hypothesis in four studies following the full-cycle approach (Mortensen & Cialdini, 2010), which involves identifying a real-world phenomenon, examining its underlying mechanism experimentally, and re-testing it in a natural setting. First, we tested Hypotheses 1 and 2 in two preregistered field studies (Studies 1a and 1b) with high external validity, conducted in the United States using Prolific. Next, we validated these results by testing Hypotheses 1–4 in a controlled, experimental setting (Study 2, preregistered) to ensure high internal validity. Finally, we returned to a natural setting, testing our model and mechanism using survey data representative of the German population to add external validity (Study 3).

To rule out alternative causal models (e.g., assuming a reverse order regarding Hypothesis 1), we also tested and compared the Akaike Information Criterion and Bayesian Information Criterion values of several alternative models. All analyses preferred and supported our hypothesized model (see Supplemental Material A for more information).

All procedures presented throughout the manuscript comply with the current American Psychological Association (APA) Ethical Principles of Psychologists and Code of Conduct. We report all manipulations, measures, and exclusions in the respective studies and follow the Journal Article Reporting Standards for Quantitative Research in Psychology (JARS; Appelbaum et al., 2018). Studies 1a, 1b, and 2 were preregistered (see links in respective sections). All data, study materials, and analysis code are available here: osf.io/pk9cs. We managed all data using the software package Stata 18 (StataCorp., 2023) and performed mediation analyses following recommendations by Preacher et al. (2007) and UCLA: Statistical Consulting Group (2024).

## Study 1a: Survey

### Participants

We followed power analysis guidelines by Fritz and MacKinnon (2007), which indicated a required sample size of 391 for the expected effects (see Supplemental Material B for details). Since the study was about political issues in the United States, we required all participants to be U.S. residents, native English speakers, and at least 21 years old. Eight participants were excluded from analysis because they failed an attention test (i.e., “please click on ‘strongly agree’ to indicate that you are still paying attention”). Two participants were dropped because they reported being less than 21 years old. Of the final 400 participants, the average age was 43 years, *SD* = 14.0; 49% were women (50% men; 1% other); 57% held a university degree. See Table 1 for a complete overview of the study variables and correlations. This study was preregistered: aspredicted.org/9pyc-n38g.pdf.^1^

**Table 1.** Correlations and Descriptive Statistics for Unstandardized Variables in Study 1a.

Variables	*M*	*SD*	%	(1)	(2)	(3)	(4)	(5)	(6)	(7)	(8)	(9)	(10)	(11)	(12)	(13)	(14)	(15)	(16)
(1) Liberalism (vs. conservatism)	4.66	1.83		1.000															
(2) Meritocracy beliefs	2.67	1.36		−.36***	1.000														
(3) Benign envy^a^	3.55	1.21		−.12*	.28***	1.000													
(4) Malicious envy	2.09	1.04		.03	−.14**	.23***	1.000												
(5) Support for redistribution	1.32	0.98		.68***	−.51***	−.04	.08	1.000											
(6) Age (in years)	43.13	14.03		−.09	.16**	−.07	−.25***	−.10*	1.000										
Gender																			
(7) Female			49	.08	−.04	−.12*	−.09	.06	.08	1.000									
(8) Male			50	−.10*	.06	.11*	.09	−.09	−.06	−.98***	1.000								
(9) Other			1	.12*	−.07	.03	.01	.14**	−.10	−.10	−.10*	1.000							
(10) Household income^b^	6.10	3.08		−.06	.14**	.14**	.04	−.20***	−.14**	−.03	.04	−.06	1.000						
(11) University degree^c^			57	.02	.03	.15**	.09	−.01	.03	.04	−.02	−.06	.37***	1.000					
(12) Unemployed^d^			8	.06	−.02	−.02	.08	.05	−.12*	.02	−.01	−.03	−.11*	−.08	1.000				
(13) Republican^d^			20	−.62***	.27***	.09	−.01	−.50***	.10*	−.02	.03	−.05	.11*	−.02	−.04	1.000			
(14) Democrat^d^			45	.58***	−.14**	.05	.06	.43***	−.01	.04	−.03	−.04	.03	.14**	.01	−.47***	1.000		
(15) Married^e^			40	−.14**	.15**	.04	−.08	−.18***	.17***	−.02	.03	−.08	.39***	.14**	−.15**	.21***	−.03	1.000	
(16) Support for redistribution (alternative scale)	4.33	2.46		.41***	−.31***	.01	.01	.56***	.02	−.02	.01	.06	−.10	.10	.05	−.28***	.25***	−.06	1.000

*Note. N* = 400.

a Measured with the respective subscale of the BeMaS (5 items, ranging from 1–6; α = .89; Lange & Crusius, 2015a).

b Monthly household gross income; measured in 10 categories/steps of $10,000, ranging from 1 (less than $15,000) to 10 (more than $95,000).

c No university degree = 0, bachelor’s degree or higher = 1.

d No = 0, yes = 1.

e Not married = 0, married = 1.

*** *p* < .001. **p < .01. *p < .05.

### Materials

#### Conservatism/Liberalism

Following seminal research (Napier & Jost, 2008), we measured conservatism (vs. liberalism) with a scale asking participants to indicate how liberal/conservative they would rate themselves on a scale from 1 (*very conservative*) to 7 (*very liberal*).

#### Dispositional Malicious Envy

We measured dispositional malicious envy with the respective subscale of the Dispositional Benign and Malicious Envy Scale (BeMaS; Lange & Crusius, 2015a, 2024). This subscale consists of five items, using a 6-point scale ranging from 1 (*strongly disagree*) to 6 (*strongly agree*). An example Item: “I wish that superior people lose their advantage” (α = .88).

#### Meritocracy Beliefs

We measured participants’ meritocracy beliefs with the “hard work” subscale of the PWE scale (McHoskey, 1994; Mirels & Garrett, 1971; Mudrack et al., 1999). This subscale consists of four items, using a 7-point scale ranging from 1 (*I disagree strongly*) to 7 (*I agree strongly*). Example item: “Anyone who is able and willing to work hard has a good chance of succeeding” (α = .87).

#### Support for Redistribution

We measured support for redistribution with a widely used scale developed by Sznycer et al. (2017; who built on Petersen et al., 2013). The instrument asks participants for their agreement on 11 statements regarding economic redistribution, using a scale ranging from 1 (*strongly disagree*) to 5 (*strongly agree*). An example item: “The wealthy should give more money to those who are worse off” (α = .93). We also included a separate scale (created by us) which measured support for redistribution by asking participants to choose between different taxation scenarios (α = .92). This allowed us to additionally capture a decision in an applied context rather than a mere evaluative judgment. All results and conclusions presented here remain largely unchanged when using this alternative scale (more details in Supplemental Material C).

#### Covariates

We controlled for several, potentially confounding variables that are known to be related to political ideology and/or dispositional envy (Harris & Henniger, 2013): household income, education level, age, and gender. Results excluding covariates did not differ substantially and can be found in Supplemental Material D.

### Results

#### Hypothesis 1: Indirect Effect of Liberalism on Malicious Envy via Meritocracy Beliefs

To test whether liberalism predicts malicious envy indirectly via reduced meritocracy beliefs, we conducted mediation analyses using 10,000 bootstrap samples and 95% bias-corrected confidence intervals (Hayes, 2018), controlling for household income, education, age, and gender. These models quantify how much liberalism predicts meritocracy beliefs (path *a*), which in turn predict the outcome (path *b*), with an indirect effect considered present when the confidence interval for *a* × *b* excludes zero (see Supplemental Material E for more details on the method).

Regression analyses revealed a significant negative effect of liberalism on meritocracy beliefs, *b* = −0.46, *p* < .001, and a significant negative effect of meritocracy beliefs on malicious envy, *b* = −0.12, *p* = .036. The total effect of liberalism on malicious envy was not significant (*b* = 0.01, *p* = .809), nor was the direct effect (i.e., controlling for the mediator: *b* = −0.03, *p* = .536). However, consistent with Hypothesis 1, there was a significant *indirect* effect of liberalism on malicious envy through meritocracy beliefs, *b* = 0.06, 95% CI [0.007, 0.12], a medium-sized effect (Kenny, 2024; Preacher & Kelley, 2011). In line with our expectations, these results point toward an indirect-only effect (Zhao et al., 2010).

#### Sequential Mediation Effect of Liberalism on Support for Redistribution via Meritocracy Beliefs and Envy

We next examined whether malicious envy subsequently predicts support for redistribution, consistent with past work (Lin & Bates, 2021; Sznycer et al., 2017). However, this was not the case: *b* = 0.02, *p* = .556. Consistently, a sequential mediation analysis (liberalism → meritocracy beliefs → malicious envy → support for redistribution) was not significant, *b* = −0.001, 95% CI [−0.007, 0.002].

#### Relative Effects of Meritocracy Beliefs and Malicious Envy on Redistribution

To further evaluate the relative contribution of meritocracy beliefs and malicious envy, both variables were entered simultaneously into regression models predicting support for redistribution. When included together, meritocracy beliefs significantly negatively predicted support for redistribution (*b* = −0.46, *p* < .001), whereas malicious envy did not (*b* = 0.005, *p* = .908, see Table 2 for comparison of models including and excluding meritocracy beliefs). A Wald test indicated that the effect of malicious envy on support for redistribution (η² = .00003) was significantly weaker than the negative effect of meritocracy beliefs (η² = .22): *F*(1, 383) = 62.66, *p* < .001.

**Table 2.** Study 1a: Relationships of Meritocracy Beliefs, Dispositional Malicious Envy, and Control Variables With Support for Redistribution.

Variable	Model 1 (excluding meritocracy beliefs)					Model 2 (including meritocracy beliefs)				
	*B*	*SE*	*t*	*P*	95% CI	*b*	*SE*	*t*	*p*	95% CI
Constant	2.16	0.21	10.48	<.001	[1.76, 2.57]	1.78	0.19	9.55	<.001	[1.41, 2.14]
Meritocracy beliefs	–					−0.46	0.04	−10.53	<.001	[−0.54, −0.37]
Dispositional malicious envy	0.05	0.05	1.05	.295	[−0.05, 0.15]	0.005	0.04	0.12	.908	[−0.08, 0.09]
Age (in years)	−0.009	0.004	−2.47	.014	[−0.02, −0.002]	−0.004	0.003	−1.15	.251	[−0.01, 0.002]
Gender										
Female^a^	0	(omitted)				0	(omitted)			
Male^a^	0.16	0.10	−1.70	.091	[−0.35, 0.03]	−0.11	0.09	−1.28	.203	[−0.28, 0.06]
Other^a^	1.07	0.48	2.23	.026	[0.13, 2.01]	0.88	0.42	2.06	.040	[0.04, 1.71]
Household income^b^	−0.08	0.02	−4.48	<.001	[−0.11, −0.04]	−0.05	0.02	−3.38	.001	[−0.08, −0.02]
University degree^c^	0.13	0.10	1.25	.214	[−0.08, 0.33]	0.11	0.09	1.24	.216	[−0.07, 0.29]

*Note.* Total *N* = 392. CI = confidence interval.

a No = 0, yes = 1.

b Measured in 10 categories/steps of $10,000, ranging from 1 (less than $15,000) to 10 (more than $95,000).

c No university degree = 0, bachelor’s degree or higher = 1.

Notably, the present findings mirror earlier work showing small zero-order associations between (malicious) envy and redistribution (Lin & Bates, 2021; Sznycer et al., 2017): In our data, too, malicious envy displayed a weak positive correlation with redistribution, which was not significant, but close to the .05 threshold (*r* = .08, *p* = .112). Crucially, once meritocracy beliefs were included in the model, the association between envy and redistribution was reduced to near zero (see Table 2 for comparisons including control variables). This pattern suggests that the small effects previously attributed to malicious envy may, in fact, reflect underlying merit judgments; specifically, perceptions that the wealthy do not deserve their advantages. This conclusion led us to preregister and test Hypothesis 2 in subsequent studies: the effect of malicious envy on support for redistribution should be smaller than the effect of meritocracy beliefs.

## Study 1b: Survey

The goal of this study was to replicate the results of Study 1a and test Hypotheses 1 and 2. This study was also preregistered: aspredicted.org/y24b-vn8g.pdf.

### Participants

To increase power and check for the possibility that results from Study 1a were attributable to chance, we decided to double the sample size, which, according to Preacher et al. (2007), would lead to a power of approximately 0.95. Again, we required all participants to be U.S. residents, native English speakers, and at least 21 years old. Six participants were excluded from the analysis because they failed an attention test. One participant was dropped because they reported being below 21 years old. Of the remaining 793 participants, the average age was 42 years, *SD* = 13.8; 49% were women (50% men; 1% other); 58% held a university degree. See Table 3 for a complete overview of the study variables and correlations.

**Table 3.** Correlations and Descriptive Statistics for Unstandardized Variables in Study 1b.

Variables	*M*	*SD*	%	(1)	(2)	(3)	(4)	(5)	(6)	(7)	(8)	(9)	(10)	(11)	(12)	(13)	(14)	(15)
(1) Liberalism (vs. conservatism)	4.68	1.75		1.000														
(2) Meritocracy beliefs	2.74	1.32		−.40***	1.000													
(3) Benign envy	3.59	1.27		−.12***	.31***	1.000												
(4) Malicious envy	2.10	1.05		−.03	−.16***	.21***	1.000											
(5) Support for redistribution	1.31	0.94		.66***	−.45***	−.16***	.07	1.000										
(6) Age (years)	41.88	13.81		−.14***	.11**	−.13***	−.28***	−.15***	1.000									
Gender																		
(7) Female			49	.09*	−.01	−.06	−.09*	.03	.09*	1.000								
(8) Male			50	−.11**	.04	.06	.08*	−.05	−.08*	−.98***	1.000							
(9) Other			1	.12**	−.12***	−.04	.03	.10**	−.07	−.09*	−.09*	1.000						
(10) Household income^a^	6.20	3.00		−.02	.12***	.11**	−.05	−.16***	.06	.04	−.03	−.06	1.000					
(11) University degree^b^			58	.04	.07*	.11**	.07	−.06	−.05	.03	−.03	−.02	.31***	1.000				
(12) Unemployed^c^			7	.05	−.09**	−.04	.03	.05	−.08*	−.10**	.11**	−.02	−.22***	−.10**	1.000			
(13) Republican^c^			19	−.67***	.29***	.06	−.03	−.48***	.14***	−.003	.01	−.04	.06	.01	−.07	1.000		
(14) Democrat^c^			46	.63***	−.19***	−.02	.07	.42***	−.06	.12**	−.12**	.01	.003	.07	−.01	−.45***	1.000	
(15) Married^d^			42	−.14***	.09**	.04	−.04	−.18***	.25***	.06	−.06	.01	.40***	.06	−.11**	.14***	−.07*	1.000

*Note. N* = 793.

a Monthly household gross income; measured in 10 categories/steps of $10,000, ranging from 1 (less than $15,000) to 10 (more than $95,000).

b No university degree = 0, bachelor’s degree or higher = 1.

c No = 0, yes = 1.

d Not married = 0, married = 1.

*** *p* < .001. **p < .01. *p < .05.

### Materials

All variables, covariates, and scales were identical to Study 1a. Results without covariates did not differ substantially and can be found in Supplemental Material D.

### Results

#### Hypothesis 1: Indirect Effect of Liberalism on Malicious Envy via Meritocracy Beliefs

Similar to Study 1a, regression analyses revealed a significant negative effect of liberalism on meritocracy beliefs, *b* = −0.52, *p* < .001, and a significant negative effect of meritocracy beliefs on malicious envy, *b* = −0.17, *p* < .001. Again, there was no total effect of liberalism on malicious envy, *b* = −0.009, *p* = .818. Interestingly, the direct effect of liberalism on malicious envy (controlling for meritocracy beliefs) was significant and opposite in sign (*b* = −0.08, *p* = .045), pointing toward competitive mediation (Zhao et al., 2010) and thus the possibility of additional, undiscovered mediators. Importantly, again consistent with Hypothesis 1, there was a significant *indirect* effect of liberalism on malicious envy via meritocracy beliefs, *b* = 0.09, 95% CI [0.05, 0.14], a medium-sized effect (Kenny, 2024; Preacher & Kelley, 2011).

#### Hypothesis 2: Meritocracy Beliefs Predict Support for Redistribution More Strongly Than Malicious Envy

Consistent with Study 1a, as well as previous work (Lin & Bates, 2021; Sznycer et al., 2017), malicious envy showed a small, positive correlation with support for redistribution, which was nonsignificant, albeit close to the .05 threshold: *r*(791) = .07, *p* = .063. When meritocracy beliefs and malicious envy were entered simultaneously into a regression model predicting support for redistribution, only meritocracy beliefs significantly predicted support (*b* = −0.41, *p* < .001). In contrast, malicious envy did not (*b* = 0.04, *p* = .252, Table 4). A Wald test confirmed that the effect of malicious envy on support for redistribution (η² = .002) was significantly weaker than the negative effect of meritocracy beliefs (η² = .19): *F*(1, 772) = 83.00, *p* < .001. Thus, Hypothesis 2 was supported. In summary, Study 1b closely replicated the pattern observed in Study 1a.

**Table 4.** Study 1b: Relationships of Meritocracy Beliefs, Dispositional Malicious Envy, and Control Variables With Support for Redistribution.

Variable	Model 1 (excluding meritocracy beliefs)					Model 2 (including meritocracy beliefs)				
	*B*	*SE*	*t*	*P*	95% CI	*b*	*SE*	*t*	*P*	95% CI
Constant	2.08	0.14	15.19	<.001	[1.81, 2.34]	1.88	0.12	15.15	<.001	[1.64, 2.13]
Meritocracy beliefs	–					−0.41	0.03	−13.27	<.001	[−0.47, −0.35]
Dispositional malicious envy	0.02	0.03	0.62	.536	[−0.05, 0.09]	−0.04	0.03	−1.15	.252	[−0.10, 0.03]
Age (in years)	−0.01	0.003	−3.84	<.001	[−0.01, −0.005]	−0.008	0.002	−3.37	.001	[−0.01, −0.003]
Gender										
Female^a^	0	(omitted)				0	(omitted)			
Male^a^	−0.13	.07	−1.88	.060	[−0.26, 0.005]	−0.09	0.06	−1.55	.121	[−0.21, 0.02]
Other^a^	0.80	.38	2.08	.038	[0.04, 1.54]	0.30	0.35	0.87	.384	[−0.38, 0.98]
Household income^b^	−0.04	.01	−3.80	<.001	[−0.07, −0.02]	−0.03	0.01	−3.06	.002	[−0.05, −0.01]
University degree^c^	−.05	.07	−0.77	.441	[−0.19, 0.08]	−0.01	0.06	−0.20	.843	[−0.14, 0.11]

*Note.* Total *N* = 780. CI = confidence interval.

a No = 0, yes = 1.

b Measured in 10 categories/steps of $10,000, ranging from 1 (less than $15,000) to 10 (more than $95,000).

c No university degree = 0, bachelor’s degree or higher = 1.

## Study 2: Experiment

The goals of this study were to test Hypotheses 1 and 2 in an experimental setting and a different context. Furthermore, we aimed to provide causal evidence for our general assumption that liberals’, but also generally, support for redistribution is largely driven by meritocracy beliefs, and less driven by (malicious) envy, as suggested by previous studies (Lin & Bates, 2021, 2024; Sznycer et al., 2017). To test this assumption experimentally, we manipulated perceived deservingness of wealth with a hypothetical scenario. We predicted that, in an ambiguous situation, liberals are especially likely to believe that wealth is *not* the deserved result of hard work, which then predicts their support for redistribution and is positively related to malicious envy. However, when, in a hypothetical meritocracy scenario, participants are made to believe that wealth is deserved, ideology should be less predictive of deservingness beliefs: Accordingly, the relationship between liberalism and deservingness beliefs should be reduced, which should further diminish the latter’s relationship with malicious envy and support for redistribution. This study was also preregistered: aspredicted.org/5fss-wrmm.pdf.

### Participants

Similar to Study 1b, we aimed to recruit 800 participants (from Prolific), which, according to Preacher et al. (2007), would lead to a power of approximately 0.95 when testing for an indirect effect. Again, we required all participants to be U.S. residents, native English speakers, and at least 21 years old. Six participants were excluded from the analysis because they failed an attention test. Of the remaining 794 participants, the average age was 42 years, *SD* = 13.7; 50% were women (48% men; 2% other); 57% held a university degree. See Table 5 for a complete overview of the study variables across conditions.

**Table 5.** Study 2: Descriptive Statistics for Variables Across Conditions.

Baseline characteristic	Control condition				Deservingness condition				Total			
	*M*	*SD*	*n*	%	*M*	*SD*	*n*	%	*M*	*SD*	*n*	%
Liberalism (vs. conservatism)	4.82	1.89	396		4.69	1.86	398		4.75	1.87	794	
Deservingness perception	4.65	1.34	396		6.25	0.85	398		5.45	1.38	794	
State benign envy	4.10	1.51	396		4.44	1.47	398		4.27	1.50	794	
State malicious envy	2.02	1.34	396		1.64	1.06	398		1.83	1.22	794	
Support for redistribution	3.31	1.25	396		2.96	1.20	398		3.13	1.24	794	
Age (in years)	41.75	13.74	395		42.09	13.59	398		41.92	13.66	793	
Gender			392				397				789	
Female^a^			204	52			190	48			394	50
Male^a^			178	45			200	50			378	48
Other^a^			10	3			7	2			17	2
Household income^b^	6.53	3.08	385		6.43	3.18	392		6.48	3.13	777	
University degree^c^			225	57			229	58			454	57
Unemployed^a^			32	8			33	8			65	8
Republican^a^			84	21			86	22			170	21
Democrat^a^			204	52			188	47			392	49
Married^d^			172	43			194	49			366	46

*Note. N* = 794.

a No = 0, yes = 1.

b Monthly household gross income; measured in 10 categories/steps of $10,000, ranging from 1 (less than $15,000) to 10 (more than $95,000).

c No university degree = 0, bachelor’s degree or higher = 1.

d Not married = 0, married = 1.

### Procedure

After agreeing to a study consent form and reading a short welcome note, the participants filled in the scale measuring liberalism (vs. conservatism). Then, the participants were randomly assigned to one of two conditions, either (a) the control condition (where deservingness/meritocracy was ambiguous) or (b) the deservingness/meritocracy condition, see below. After the manipulation, participants were asked to complete the scales measuring the mediator variable (i.e., participants’ perceptions of merit/deservingness, McHoskey, 1994; Mirels & Garrett, 1971; Mudrack et al., 1999). Afterwards, we measured state malicious envy (Lange, Paulhus, et al., 2018). This was followed by a scale measuring the dependent variable, support for redistribution (Hansen, 2023), and a survey of demographics (household income, education level, age, gender, employment status, marital status, and political party affiliation). Finally, participants were redirected to Prolific.

### Materials

#### Manipulation of Perceived Deservingness

Following previous research (Hansen, 2023; Jetten et al., 2015; Neerdaels et al., 2024), we manipulated perceived deservingness with a hypothetical societal scenario (see Supplemental Material F for full texts). More specifically, similar to Hansen (2023), we instructed participants to imagine living in a small village. In both conditions, we introduced a villager named “Alex” (whose gender remained unspecified), who “is one of the wealthiest people in the village.” In the (ambiguous) control condition, no more information is given. In the deservingness condition, we highlight that Alex’s wealth has been well deserved: “(. . .) success has not come easily for Alex. Alex has always been a very hardworking person, and this has made Alex rich. Therefore, you and everyone you know agree that Alex’s success is well deserved.”

Thus, importantly, we manipulate meritocratic thinking at a situational level (namely, participants’ judgments of whether a hypothetical person deserves their wealth), rather than assessing endorsement of meritocracy as a broad ideological worldview. We treat these deservingness judgments as a contextualized expression of meritocracy beliefs, consistent with the view that meritocratic ideology provides the evaluative lens through which individuals infer whether specific outcomes reflect effort and ability.

Because malicious envy typically arises when advantages are seen as undeserved, shifts in perceived deservingness are expected to influence both merit-related appraisals and envy, albeit not in identical ways. Both constructs are shaped by multiple psychological processes, and deservingness information represents only one of several possible causes. Accordingly, the experiment was not designed to isolate these constructs but to demonstrate that changing perceived deservingness simultaneously shifts envy and redistribution attitudes in theoretically consistent directions.

To rule out the possibility that the effects of the manipulation on, for example, support for redistribution are attributable to the fact that Alex may appear more likable in the deservingness condition, we ran several additional analyses (using the malicious envy scale as a covariate; see Supplemental Material G), all of which indicated no unintended confounding.

#### Conservatism/Liberalism

Similar to Studies 1a and 1b, we measured liberalism (vs. conservatism) with a scale asking participants to indicate how liberal/conservative they would rate themselves on a scale from 1 (*very conservative*) to 7 (*very liberal*).

#### Perceptions of Deservingness

Similar to previous Studies 1a and 1b, we measured participants’ situational perception of deservingness with an adapted version of the “hard-work” subscale of the PWE scale used in Studies 1a and 1b (McHoskey, 1994; Mirels & Garrett, 1971; Mudrack et al., 1999; η = .96), using a 7-point scale ranging from 1 (*I disagree strongly*) to 7 (*I agree strongly*). An example item: “Alex succeeded because she or he was able and willing to work hard for it.” We interpret these perceptions as a situational manifestation of meritocratic belief processes.

#### Support for Redistribution

We measured support for redistribution with five items, building on Hansen (2023) and Sznycer et al. (2017). Thus, similar to Studies 1a and 1b, this instrument asked participants for agreement on statements regarding economic redistribution, using a scale ranging from 1 (*strongly disagree*) to 5 (*strongly agree;* η = .95). An example item: “The village should take more money from people like Alex to give to the poor.”

#### State Malicious Envy

We measured state malicious envy with the respective subscale of the Pain Driven Dual Envy (PaDE) Theory State Envy Scale (Lange, Paulhus, et al., 2018), which, again, we adapted to match the present experimental context. This subscale consists of four items ranging from 1 (*does not apply at all*) to 7 (*applies very much*). An example item: “I would feel hatred because Alex is richer than me” (η = .91).

### Results

#### Manipulation Check

A *t*-test confirmed that the deservingness manipulation was successful: Participants in the deservingness condition believed more strongly that Alex’s success was merited (*M* = 6.25, *SD* = 0.85) than those in the ambiguous condition (*M* = 4.65, *SD* = 1.34), *t*(792) = −20.08, *p* < .001.

#### Preliminary Analyses

We expected liberalism to predict lower perceptions of deservingness, particularly in the ambiguous condition, and for these beliefs to relate to malicious envy and support for redistribution. As predicted, a significant interaction emerged between liberalism and the experimental manipulation. In the control condition, liberals were less likely than conservatives to view Alex’s success as deserved (*b* = 0.28, *p* < .001, η² = .02). Simple-slope analyses indicated that liberalism was negatively associated with deservingness perceptions in the ambiguous condition (*b* = −0.52, *p* < .001, β = .38), but less so in the deservingness condition (*b* = −0.25, *p* < .001, β = .18; see Figure 1).

**Figure 1.**
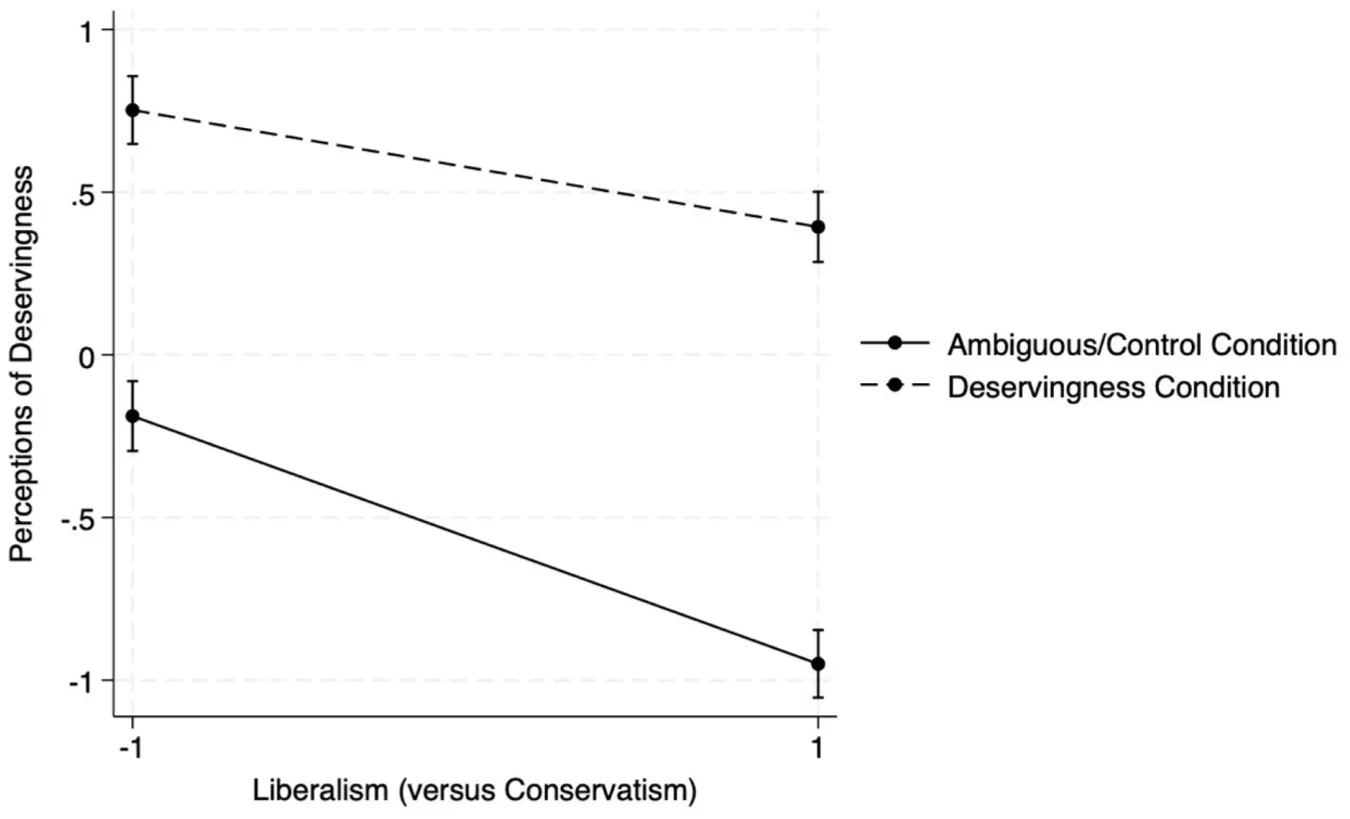
Study 2: Interaction effect of liberalism (vs. conservatism) and experimental manipulation (ambiguous/control vs. deservingness condition) on perceptions of deservingness. *Note.* Scales measuring perceptions of deservingness and liberalism are centered; Bars indicate 95% confidence intervals.

#### Hypothesis 1: Indirect Effect of Liberalism on Malicious Envy via Meritocracy Beliefs/Perceptions of Deservingness, Moderated by Deservingness Condition

We next tested whether liberals were more likely to report malicious envy indirectly through reduced deservingness perceptions, especially in the ambiguous condition. Results indicated a general causal effect of deservingness perceptions on malicious envy. Participants in the deservingness condition reported less malicious envy (*M* = 1.64, *SD* = 1.06) than those in the ambiguous condition (*M* = 2.02, *SD* = 1.34), *t*(792) = 4.45, *p* < .001.

To assess whether the hypothesized indirect path varies by condition, we computed the Index of Moderated Mediation (IMM; Hayes, 2018). Conceptually, the IMM evaluates whether the strength of the indirect effect of liberalism on the outcome (e.g., malicious envy or redistribution support) via deservingness perceptions varies between the deservingness and ambiguous conditions. This approach differs from subsequent regression-based indirect effect estimates, which quantify indirect paths without considering moderation by the manipulation. The IMM was significant (*b* = −0.07, 95% CI [−0.12, −0.03]), indicating that the indirect effect of liberalism on malicious envy was moderated by condition.

In the ambiguous condition, the indirect effect was significant (*b* = 0.13, 95% CI [0.09, 0.19]); in the deservingness condition, the effect was smaller but still significant (*b* = 0.06, 95% CI [0.04, 0.09]). The direct effect (of liberalism on malicious envy) was insignificant (*b* = 0.01, *p* = .780). Together, these results support Hypothesis 1.

#### Hypothesis 2: Meritocracy Beliefs/Deservingness Perceptions Predict Support for Redistribution More Strongly Than Malicious Envy

To compare predictive strength, we regressed support for redistribution on malicious envy and deservingness perceptions while controlling for income, education, age, and gender (Table 6).

**Table 6.** Study 2: Relationships of Deservingness Perceptions, Malicious Envy, and Control Variables With Support for Redistribution.

Variable	Model 1 (excluding deservingness perceptions)					Model 2 (including deservingness perceptions)				
	*b*	*SE*	*t*	*p*	95% CI	*b*	*SE*	*T*	*p*	95% CI
Constant	3.65	0.19	19.13	<.001	[3.27, 4.02]	3.75	0.17	22.46	<.001	[3.43, 4.08]
Deserv. percept.	–					−0.41	0.04	−9.66	<.001	[−0.50, −0.33]
State malicious envy	0.32	0.04	7.54	<.001	[0.24, 0.41]	0.19	0.04	4.44	<.001	[0.11, 0.28]
Age (in years)	−.01	0.003	−3.61	<.001	[−0.02, −0.005]	−0.01	0.003	−3.34	.001	[−0.02, −0.004]
Gender										
Female^a^	−0.05	0.08	−0.64	.523	[−0.22, 0.11]	−0.01	0.08	−0.18	.855	[−0.17, 0.14]
Male^a^	(omitted)					0	(omitted)			
Other^a^	0.83	0.29	2.86	.004	[0.26, 1.39]	0.56	0.28	2.03	.042	[0.02, 1.11]
Household income^b^	−.05	.01	−3.32	.001	[−0.08, −0.02]	−0.04	0.01	−2.86	.004	[−0.07, −0.01]
University degree^c^	0.08	0.03	2.46	.014	[0.02, 0.15]	0.08	0.09	0.98	.330	[−0.09, 0.25]

*Note.* Total *N* = 774. CI = confidence interval.

a No = 0, yes = 1.

b Measured in 10 categories/steps of $10,000, ranging from 1 (less than $15,000) to 10 (more than $95,000).

c No university degree = 0, bachelor’s degree or higher = 1.

A Wald test showed that the effect of malicious envy on support for redistribution (η² = .02) was significantly weaker than the negative effect of deservingness perceptions (η² = −.11): *F*(1, 770) = 148.02, *p* < .001. Results mirrored those without covariates (Supplemental Material B). Thus, Hypothesis 2 was supported.

#### Hypothesis 3: Interaction of Liberalism and Condition on Support for Redistribution

We predicted that liberalism would more strongly predict redistribution support in the ambiguous condition than in the deservingness condition. Although liberalism robustly predicted support for redistribution overall (*b* = 0.66, *p* < .001, β = .54), the hypothesized interaction with the manipulation was not significant (*b* = 0.02, *p* = .830). Thus, Hypothesis 3 was not supported.

Importantly, however, this null effect is not inconsistent with our broader theoretical account: Hypotheses 2 and 4 posit that deservingness (i.e., meritocracy) beliefs should play a central role in shaping redistribution attitudes. Under this view, deservingness beliefs should predict support for redistribution generally, not only among liberals. Supporting this, participants in the deservingness condition reported significantly lower support for redistribution (*M* = 2.96, *SD* = 1.19) than those in the ambiguous condition, irrespective of political leaning (*M* = 3.31, *SD* = 1.25), *t*(792) = 4.07, *p* < .001. Although not preregistered, this causal effect aligns with the idea that when wealth is perceived as deserved, individuals, regardless of ideology, are more likely to oppose redistribution, as indicated by earlier findings (Hoogland, 2016).

#### Hypothesis 4: Moderated Indirect Effect of Liberalism on Support for Redistribution via Meritocracy Beliefs/Deservingness Perceptions

Finally, we examined whether the indirect effect of liberalism on support for redistribution via deservingness perceptions was moderated by the deservingness manipulation. The IMM was significant (*b* = −0.06, 95% CI [−0.10, 0.03]), indicating moderated mediation. In the ambiguous condition, there was a medium-sized indirect effect (*b* = 0.11, [0.07, 0.14]); in the deservingness condition, the effect was smaller (but also significant; *b* = 0.05, [0.03, 0.07]). Thus, Hypothesis 4 was supported. Together, the findings of Study 2 support Hypotheses 1, 2, and 4 and thus replicate the pattern from Studies 1a/b in an experimental setting.

## Study 3: GESIS Panel

The goals of this study were to test Hypotheses 1 and 2 in a different, that is, German population, thus adding external validity to our claims. To do so, we analyzed data from the GESIS panel, a panel-based social science survey using random samples of the adult German-speaking population of Germany (GESIS, 2025). Respondents can answer web-based or by article questionnaires. Data for the GESIS panel are collected in three to four waves per year and are representative of the adult German population (GESIS, 2025). Data and codebook can be downloaded via: doi.org/10.4232/1.13476.

Given that some key variables (e.g., envy) were measured only once but at different time points in the same participant sample, we employed a time-lagged design using temporally separated measurements collected between 2013 and 2017. Although the data therefore do not support traditional longitudinal modeling (e.g., cross-lagged panel analyses), we treated them as temporally ordered observations and conducted cross-sectional analyses that take advantage of the time lag between measurement points. This approach allows us to examine, for example, how earlier ideological beliefs (meritocracy beliefs measured in 2014) predict envy dispositions (measured in 2015). Analyzing the data in this time-ordered manner strengthens causal inference relative to a purely cross-sectional design, even if the data do not allow for fully longitudinal modeling.

### Participants

The sample size was 2,183 for our variables of interest. The average age was 47 years, *SD* = 14.1; 52% were women; 29% held a university degree. See Table 7 for a complete overview of the study variables and correlations.

**Table 7.** Correlations and Descriptive Statistics for Unstandardized Variables in Study 3.

Variable	*M*	*SD*	%	1	2	3	4	5	6	7	8
(1) Support for redistribution	3.93	1.01		1.00							
(2) Liberalism (vs. conservatism)	5.42	1.95		.23***	1.00						
(3) Meritocracy beliefs	2.94	1.12		−.18***	−.12***	1.00					
(4) Dispositional envy	2.21	1.01		−.01	.02	−.04*	1.00				
(5) Age in years	46.96	14.08		.10***	−.03*	−.13***	−.30***	1.00			
(6) Gender: female			52	.02	.06***	−.08***	.00	.02	1.00		
(7) Household income^a^	9.86	2.76		−.19***	−.03	.18***	−.03	.03*	−.10***	1.00	
(8) University degree^b^			29	−.14***	.02	.10***	.02	.06***	−.04***	.34***	1.00

*Note. N* = 2,673.

a Monthly household gross income; scale ranging from 1 (less than €700) to 14 (€6,000 or more).

b No university degree = 0, university degree = 1.

*** *p* < .001. *p < .05.

### Materials

#### Liberalism

Liberalism (vs. conservatism) was measured with a scale asking participants to indicate how right-wing/left-wing they would rate themselves on a scale from 0 (*right-wing*) to 10 (*left-wing*).

#### Meritocracy Beliefs

Participants’ meritocracy beliefs were measured with an item from a battery designed to measure attitudes toward social differences (reverse coded): “What one gets in life depends not so much on one’s own efforts but on the economic situation, the situation on the employment market, wage agreements, and the social benefits provided by the state.”

#### Support for Redistribution

Support for redistribution was measured with the following item: “The government should take measures to reduce income disparities.”

#### Dispositional Envy

We measured dispositional envy with the 15-item Domain-Specific Envy Scale (DSES; Rentzsch & Gross, 2015), which assesses envy across competence, attractiveness, and wealth (1 = *does not apply at all* to 10 = *applies completely*; α = .93). Because wealth-related envy is especially theoretically relevant here, we also reran all analyses using the 5-item wealth subscale (α = .85; e.g., “It bothers me when others own things that I cannot have”), which produced comparable results (Supplemental Material H).

Generally, prior work shows that the DSES correlates strongly with malicious envy but only weakly with benign envy (Lange, Blatz, et al., 2018), making it an appropriate proxy for the malicious envy measures used in Studies 1a/b and 2.

#### Covariates

Similar to Studies 1a and 1b, we controlled for household income, education level, age, and gender.^2^

### Results

#### Hypothesis 1: Indirect Effect of Liberalism on Envy via Meritocracy Beliefs

Similar to Studies 1a and 1b, regression analyses revealed a significant negative effect of liberalism on meritocracy beliefs, *b* = −0.06, *p* < .001, and a significant negative effect of meritocracy beliefs on envy, *b* = −0.08, *p* < .001. Again, there was neither a total (*b* = −0.03, *p* = .177) nor a direct effect of liberalism on envy (*b* = −0.04, *p* = .101). However, again supporting Hypothesis 1, there was an *indirect* effect of liberalism on envy via meritocracy beliefs, *b* = 0.005, 95% CI [0.003, 0.01], although the effect was small (Kenny, 2024; Preacher & Kelley, 2011).

#### Hypothesis 2: Meritocracy Beliefs Versus Envy as Predictors of Support for Redistribution

We then examined whether meritocracy beliefs predicted support for redistribution more strongly than envy. Meritocracy beliefs significantly predicted support for redistribution (*b* = −0.17, *p* < .001), whereas envy did not (*b* = 0.005, *p* = .869; see Table 8 for a comparison of models including and excluding meritocracy beliefs). A Wald test confirmed that the effect of envy on support for redistribution (η² = .0.0000005) was significantly weaker than the negative effect of meritocracy beliefs (η² = .03): *F*(1, 1596) = 23.84, *p* < .001.

**Table 8.** Study 3: Relationships of Meritocracy Beliefs, Dispositional Envy, and Control Variables With Support for Redistribution.

Variable	Model 1 (excluding meritocracy beliefs)					Model 2 (including meritocracy beliefs)				
	*b*	*SE*	*t*	*P*	95% CI	*b*	*SE*	*T*	*p*	95% CI
Constant	4.29	0.12	34.82	<.001	[4.05, 4.53]	4.36	0.14	30.92	<.001	[4.08, 4.63]
Meritocracy beliefs	–					−0.17	0.03	−6.24	<.001	[−0.22, −0.11]
Dispositional envy	−0.0004	0.03	−0.01	.989	[−0.05, 0.05]	0.005	0.03	0.17	.869	[0.05, 0.06]
Age (in years)	0.006	0.002	3.36	.001	[0.002, 0.009]	0.003	0.002	1.65	.098	[−0.0006, 0.007]
Gender										
Female	0.01	.04	0.29	.770	[−0.07, 0.10]	0.07	0.05	1.33	.185	[−0.03, 0.16]
Household income^a^	−0.06	0.009	−7.08	<.001	[−0.08, −0.04]	−0.06	0.01	−6.13	<.001	[−0.08, −0.04]
University degree^b^	−0.18	.05	−3.41	.001	[−0.29, −0.08]	−0.12	0.06	−2.07	.038	[−0.23, −0.006]

*Note.* Total *N* = 1,603. CI = confidence interval.

a Monthly net income, ranging from 1 (€700 or less) to 14 (€6,000 or more).

b No university degree = 0, university degree = 1.

Together, these findings support Hypotheses 1 and 2 and extend the results from Studies 1a/1b by demonstrating the same pattern in a time-lagged design.

## General Discussion

Our findings clarify how envy, liberalism, and support for redistribution relate. Drawing on system justification theory (Jost, 2020), we proposed that liberalism is indirectly linked to malicious envy because liberals are more likely to view wealth as undeserved, attributing it to, for example, luck or privilege rather than hard work. Such perceptions of unfair advantage are a key antecedent of malicious envy (Smith & Kim, 2007). Supporting our predictions, across the studies, liberals consistently endorsed meritocracy beliefs less, which in turn predicted malicious envy; yet liberalism and malicious envy were largely uncorrelated. This largely indirect pattern helps explain inconsistent prior findings (Harris & Henniger, 2013) and contradicts the “politics of envy” claim: liberals are not inherently more envious, but differ in how they judge the deservingness of wealth.

Perhaps more importantly, our findings also clarify the link between envy and support for redistribution. Although past work sometimes found small effects of malicious envy (Lin & Bates, 2021), we argue, and consistently observed across Studies 1a, 1b, and 3, that such associations largely vanish once meritocracy beliefs are taken into account. This pattern suggests that earlier correlations between envy and redistribution reflected perceived undeserved advantage rather than envy itself.

Experimental results (Study 2) strengthened this interpretation: When participants were placed in a “true meritocracy” scenario, liberals judged wealth as more deserved and, correspondingly, reported lower support for redistribution. Further supporting our claims, the experiment also showed causal effects of meritocracy beliefs on redistribution attitudes, independent of ideology, and (negatively) on malicious envy. Thus, ideological differences in redistribution preferences are better explained by merit judgments than by envy: conservatives tend to view the wealthy as deserving, whereas liberals are more sensitive to structural unfairness. However, conservatives also tend to support redistribution more when they perceive the wealthy as undeserving.

Our findings underscore the need to distinguish envy from meritocracy beliefs in research on support for redistribution and possibly in other socio-political domains. For instance, negative attitudes toward perceived high-status groups, such as in populist anti-elitism or antisemitism, may stem more from perceptions of (un)fairness than from envy (Abts & Rogenhofer, 2024; Kressel, 2024).

### Limitations

The present findings are limited in several respects. First, our conclusions about the link between liberalism and meritocracy beliefs are correlational, as political ideology is a highly stable trait that is difficult to manipulate (Sears & Funk, 1999). Nonetheless, prior work has repeatedly documented the association between conservatism and meritocracy or system-justifying beliefs (Furnham & Bland, 1983; Jost et al., 2003; Napier & Jost, 2008). Importantly, our own experimental results offer causal evidence that meritocracy beliefs shape both malicious envy and support for redistribution, providing support for the theorized direction of effects. The time-lagged data from Study 3 and consistent model comparisons further support the hypothesized direction of pathways. Still, these results do not entirely rule out the possibility of reverse effects. For example, envy could also influence fairness perceptions. Future work should examine such possibilities using experimental or longitudinal designs.

Another limitation, and a promising direction for future research, concerns our reliance on a unidimensional left–right conceptualization of ideology. Although such conceptualizations are common and valid, scholarship highlights that ideology can be multidimensional, with economic and social dimensions that may diverge (Duckitt & Sibley, 2009; Johnston & Ollerenshaw, 2020). Future work could therefore examine whether the present effects replicate when these dimensions are disentangled. For instance, economic conservatism may be especially tied to meritocratic ideology and, consequently, to lower support for redistribution.

Another direction for future research concerns the role of income. One might expect malicious envy to be most relevant among individuals lower in the socioeconomic hierarchy, raising the question of whether income moderates some of the found relationships. Exploratory analyses did not provide consistent evidence that lower-income liberals reported more malicious envy or that the latter’s link with redistribution was stronger among low-income participants (Supplemental Material I). Still, future work may examine finer contextual moderators, such as relative socioeconomic distance or local inequality, which may matter more than absolute income.

Similarly, beyond the “politics of envy” narrative, future research should examine additional arguments commonly raised in debates about redistribution, such as concerns about coercion, personal freedom, or the long-term economic consequences of redistribution. Given the scale and urgency of rising inequality, developing an accurate understanding of the beliefs that shape attitudes toward redistribution is crucial.

A further limitation is our reliance on self-report measures of redistribution. Although Study 1a included a decision task with explicit (albeit hypothetical) consequences, none of our studies used fully incentivized paradigms in which participants redistributed real money. Such approaches would offer a stronger test of redistribution preferences and their psychological drivers and thus should be incorporated in future research.

Finally, Study 2 assessed meritocratic thinking at the situational level, that is, participants’ judgments of whether Alex deserved their wealth, rather than global ideological endorsement. Thus, future work should experimentally examine whether also more general ideological beliefs shape envy and redistribution preferences.

### Direct or Indirect: Clarifying the Relationship Between Liberalism and Envy

Across studies, zero-order correlations between liberalism and envy were small and nonsignificant, and the pooled estimate was near zero (*r* ≈ .02). Detecting a relationship of this magnitude with 80% power would require a sample of roughly 20,000 participants, indicating that any total effect, if present, is exceedingly small and likely negligible in practical terms.

Consistent with our predictions, the pattern found throughout the studies reflects indirect-only mediation (Zhao et al., 2010): Liberalism predicts lower meritocracy beliefs, which in turn are associated with envy, while the remaining direct effect was small, nonsignificant, and typically opposite in sign to the indirect effect. Notably, one study showed a significant opposite-signed direct effect, indicating competitive mediation (Zhao et al., 2010). This suggests that additional, countervailing psychological processes may partially offset the meritocracy pathway, resulting in an overall near-zero total effect. Accordingly, future work may examine alternative mechanisms, such as liberals’ greater empathy or prosocial attitudes that could operate in the opposite direction (Hasson et al., 2018; Van Lange et al., 2012).

### Practical Implications

Although people may disagree on how to address inequality, most agree that action is needed, making an accurate understanding of policy motives important (Witko & Moldogaziev, 2025). Because moral framing shapes policy support (Skitka & Wisneski, 2011; Van Zant & Moore, 2015), portraying redistribution as “politics of envy” risks distorting the debate. Our results suggest this claim is misplaced: merit judgments, not envy, are the primary driver of redistribution support. We hope these insights contribute to more informed discussions about reducing inequality.

## Supplemental Material

sj-docx-1-psp-10.1177_01461672251410720 – Supplemental material for Politics of Envy? Meritocracy Beliefs, Not Envy, Drive Support for Redistribution Supplemental material, sj-docx-1-psp-10.1177_01461672251410720 for Politics of Envy? Meritocracy Beliefs, Not Envy, Drive Support for Redistribution by Jasper Neerdaels, Lisa Blatz and Jan Crusius in Personality and Social Psychology Bulletin

## Data Availability

Studies 1a, 1b, and 2 were preregistered (see links in respective sections). Study materials, data, and analysis code are available here: osf.io/pk9cs. Study materials and data used in Study 3 are publicly available on the GESIS data archive website: doi.org/10.4232/1.13476.

## Notes

1. Our initial goal in Study 1 was to test whether the hypothesized indirect effect of liberalism on malicious envy (H1) would further predict support for redistribution, thus supporting previous findings (Lin & Bates, 2021; Sznycer et al., 2017); See Supplemental Material B for power analysis. Moreover, for Studies 1a/b, & 2, we also preregistered hypotheses for a separate project examining the relationship between *conservatism*, *benign* envy, and support for redistribution (see Table 1 for correlations and additional information).

2. Results without covariates (Supplemental Material B) were largely similar, with one exception: using the full DSES (Rentzsch & Gross, 2015), the indirect effect of liberalism on envy via meritocracy beliefs was not significant. However, when controlling only for age, recommended based on Harris and Henniger (2013), the predicted indirect effect re-emerged. When using the wealth-specific DSES subscale, results without covariates were unchanged.
