# Supplementary material for: Politics of Envy? Meritocracy Beliefs, Not Envy, Drive Support for Redistribution
Source: Pers Soc Psychol Bull. 2026 Jan 8;52(12):4286–305. doi: 10.1177/01461672251410720 (PMC13736319; doi:10.1177/01461672251410720)
Supplement: Supplementary file 1 [file sj-docx-1-psp-10.1177_01461672251410720.docx]

Supplemental Online Materials

**Politics of Envy? Meritocracy Beliefs, Not Envy, Drive Support for Redistribution**

Table of Contents

[Supplemental Material A: Testing Alternative Causal Models	3](#_Toc215838023)

[Supplemental Material B: Power Analyses	3](#_Toc215838024)

[Supplemental Material C: Results Using Alternative Support for Redistribution Scale (Study 1a)	4](#_Toc215838025)

[Supplemental Material D: Results Without Covariates	5](#_Toc215838026)

[Supplemental Material E: Regression-Based Mediation Equations	7](#_Toc215838027)

[Supplemental Material F: Manipulation Texts Study 2	8](#_Toc215838028)

[Supplemental Material G: Ruling out Potential Confound (Liking) in Study 2	8](#_Toc215838029)

[Supplemental Material H: Results of Study 3 Using the Wealth-Related Envy Subscale	9](#_Toc215838030)

[Supplemental Material I: Testing Interaction Effects with Income	10](#_Toc215838031)

# **Supplemental Material A: Testing Alternative Causal Models**

To rule out alternative causal orders regarding Hypothesis 1 in our correlational studies (e.g., dispositional malicious envy predicting meritocracy beliefs, which then predicts liberalism), we tested and compared different models using Akaike Information Criterion and Bayesian Information Criterion. As Table S1 shows, the hypothesized model (liberalism → meritocracy beliefs → envy) was preferred throughout.

**Table S1**

*Model Fit Comparison Across Studies*

| Model Direction | Study 1a AIC | Study 1a BIC | Study 1b AIC | Study 1b BIC | Study 3 AIC | Study 3 BIC |
| --- | --- | --- | --- | --- | --- | --- |
| Liberalism → meritocracy → envy | 11630.94 | 11739.32 | 22949.60 | 23068.86 | 105633.4 | 105721.5 |
| Envy → meritocracy → liberalism | 11678.70 | 11787.07 | 23868.57 | 23987.83 | 105646.8 | 105734.9 |
| Envy → liberalism → meritocracy | 12147.75 | 12207.32 | 23886.25 | 23956.18 | 105653.9 | 105741.9 |

*Note.* AIC = Akaike Information Criterion; BIC = Bayesian Information Criterion. Lower values indicate better model fit.

# **Supplemental Material B: Power Analyses**

***Study 1a***

**Hypothesis 1.** To determine our target sample size, we conducted an a priori power analysis for the indirect effect. Following Fritz and MacKinnon (2007), we assumed a medium effect for the path from ideology to meritocracy beliefs (path *a*), corresponding to *r* = .39, and a small effect for the path from meritocracy beliefs to the outcome (path *b*), corresponding to *r* = .14. These assumptions were informed by previous work showing correlations of a similar magnitude between conservatism and the Protestant Work Ethic Scale (e.g., *r* = .47; Furnham & Bland, 1983) and between deservingness beliefs and redistribution-relevant judgments (e.g., *r* = .19; Van de Ven et al., 2015). Fritz and MacKinnon’s (2007) simulations indicate that a sample of approximately *N* ≈ 391 is sufficient to detect such an indirect effect. Using these values in the more recent Monte-Carlo approach implemented by Schoemann et al. (2017) yielded an estimated power of .91 for our final sample size.

**Sequential Mediation Model.** Our initial goal in Study 1a was to test whether the hypothesized indirect effect of liberalism on malicious envy (H1) would further predict support for redistribution, thus supporting previous findings (Lin & Bates, 2021; Sznycer et al., 2017). Based on an expected correlation of *r* = .25 between malicious envy and support for redistribution (as reported by Lin & Bates, 2024) and using the Monte-Carlo procedure implemented by Schoemann et al. (2017), a sample size of approximately *N* ≈ 340 was estimated to provide a power of .80.

# **Supplemental Material C: Results Using Alternative Support for Redistribution Scale (Study 1a)**

For exploratory reasons, we developed a scale measuring support for redistribution with three items (⍺ = .92). The scale presents three different, hypothetical scenarios in which the reader is a member of the middle class. Participants were asked to choose the size of their income tax on a scale ranging from 1 (2,5%) to 11 (25%). Detailed information was given on how much each option would cost the participants themselves, how much a (hypothetical) rich person would have to pay, and how much a (hypothetical) poor person would gain from each tax option. An example item:

“You have a monthly income of $5,000. There is a member of the upper class whose monthly income is $10,000. There is also a member of the lower class, whose income is $1,000. (All of you may or may not have other income streams, too.) Imagine that there is something like an income tax that you (middle class) and the person from the upper class have to pay, in order to support the member of the lower class. You get to decide how high that tax is. This means that you can decide how much you and the upper-class member will have to pay to the lower-class member. Below, there are various options of percentages that you’d have to pay. Please choose one.”

Results of analyses using this scale were similar to results of the other scale used throughout the manuscript (Sznycer et al., 2017): A regression analysis revealed a significant, positive relationship between liberalism and perceptions that success is generally *not* a deserved result of hard work (*b* = 0.47, *p* < .001), which then significantly predicted malicious envy (*b* = 0.12, *p* = .036). Meritocracy beliefs significantly predicted support for redistribution (*b* = -0.43, *p* < .001). However, again, there was no significant relationship between malicious envy and support for redistribution when using the alternative (support for redistribution) scale (*b* = -0.06, *p* = .611). Again, naturally, sequential mediation analysis yielded insignificant results (*b* = -0.003, 95% CI = [-0.02, 0.008]).

Moreover, the alternative support for redistribution scale did not correlate significantly with malicious envy *r*(400) = .01, *p* = .878, similar to the scale used in the main document.

# **Supplemental Material D: Results Without Covariates**

***Study 1a***

Supporting Hypothesis 1, results of a mediation analysis (10,000 bootstrapping samples) excluding covariates revealed a significant indirect effect of liberalism on malicious envy via meritocracy beliefs (*b* = 0.07, 95% CI = [0.02, 0.14]), which may be interpreted as a medium-sized effect (Kenny, 2024; Preacher & Kelley, 2011).

Results of a regression analysis without covariates revealed a significant, positive relationship between liberalism and perceptions that success is generally *not* a deserved result of hard work (*b* = 0.48, *p* < .001), which then significantly predicted malicious envy (*b* = 0.15, *p* = .007). There was no direct effect of liberalism (controlling for meritocracy beliefs) on malicious envy (*b* = -0.02, *p* = .658). Again, there was no significant relationship between malicious envy and support for redistribution (*b* = 0.02, *p* = .505).

Accordingly, unsurprisingly, the results of a sequential mediation analysis (10,000 bootstrapping samples) yielded insignificant results: (*b* = 0.002, 95% CI = [-0.003, 0.008]).

***Study 1b***

Supporting Hypothesis 1, results of mediation analysis (10,000 bootstrapping samples) revealed a significant indirect effect of liberalism on malicious envy via meritocracy beliefs (*b* = 0.09, 95% CI = [0.05, 0.14]), which again may be interpreted as a medium-sized effect (Kenny, 2024; Preacher & Kelley, 2011).

Results of a regression analysis without covariates revealed a significant, positive relationship between liberalism and perceptions that success is generally *not* a deserved result of hard work (*b* = 0.52, *p* < .001), which then significantly predicted malicious envy (*b* = 0.18, *p* < .001). There was no direct effect of liberalism (controlling for meritocracy beliefs) on malicious envy (*b* = -0.04, *p* = .378). Again, there was no significant relationship between malicious envy and support for redistribution (*b* = -0.04, *p* = .381).

***Study 2***

Supporting Hypothesis 2, results of a Wald test confirmed that the effect of malicious envy on support for redistribution (η2 = .04) was significantly smaller than the effect of meritocracy beliefs when no control variables were included (η2 = .12): *F*(1, 791) = 189.87, *p* < .001.

***Study 3***

Testing Hypotheses 1 and 2, we followed the same procedures as described in Studies 1 and 2. However, unlike in Studies 1a, 1b, and 2, there was no indirect effect of liberalism on dispositional envy via meritocracy beliefs (Hypothesis 1) when using the full Domain-Specific Envy Scale (DSES; Rentzsch & Gross, 2015) (*b* = 0.002, 95% CI = [-0.00001, 0.006]). There was also no direct effect of liberalism (controlling for meritocracy beliefs) on envy (*b* = -0.04, *p* = .071).

Hypothesis 1 was supported, however, when using the wealth-specific DSES subscale (Rentzsch & Gross, 2015) (*b* = 0.004, 95% CI = [0.001, 0.007]; a small-sized effect (Kenny, 2024; Preacher & Kelley, 2011)). Here, the direct effect of liberalism (controlling for meritocracy beliefs) on envy was also significant (*b* = -0.06, *p* = .003).

Moreover, when only controlling for age, a covariate that is highly recommended based on earlier results by Harris and Henniger (2013), Hypothesis 1 was supported, too (*b* = 0.006, 95% CI = [0.003, 0.009]; a small-sized effect (Kenny, 2024; Preacher & Kelley, 2011)).

In order to test Hypothesis 2 without covariates, we regressed support for redistribution on dispositional envy and deservingness beliefs. Again, there was no significant relationship between dispositional envy and support for redistribution (*b* = -0.003, *p* = .889), while meritocracy beliefs did predict support for redistribution (*b* = -0.19, *p* < .001; η^2^ = .04). Results were similar when using the wealth-specific DSES subscale (Rentzsch & Gross, 2015): There was no significant relationship between wealth related envy and support for redistribution (*b* = -0.005, *p* = .831), while meritocracy beliefs did predict support for redistribution (*b* = -0.19, *p* < .001; η^2^ = .04). Thus, Hypothesis 2 was supported.

# **Supplemental Material E: Regression-Based Mediation Equations**

To test indirect effects in the manuscript, e.g., whether liberalism predicts malicious envy indirectly via reduced meritocracy beliefs, we conducted mediation analyses using 10,000 bootstrap samples and 95% bias-corrected confidence intervals (Hayes, 2018). Conceptually, the indirect effect reflects the degree to which liberalism predicts meritocracy beliefs (path *a*), which in turn predict the outcome (path *b*), controlling for liberalism. Statistically, the indirect effect is the product (*a* × *b*) of these two unstandardized regression coefficients. To assess its precision, we used nonparametric bootstrapping, which generates a bias-corrected confidence interval that does not rely on normality assumptions. An indirect effect is considered present when this interval does not include zero.

The indirect effects reported in this manuscript were estimated using ordinary least squares (OLS) regression–based mediation models following Hayes (2018). The mediation procedure can be expressed using the following two equations:

$$\begin{matrix} & M=i_{M}+aX+e_{M} & & \end{matrix}$$

$$\begin{matrix} & Y=i_{Y}+c^{'}X+bM+e_{Y} & & \end{matrix}$$

where

- $X$= predictor (e.g., liberalism)
- $M$= mediator (e.g., meritocracy beliefs)
- $Y$= outcome (e.g., envy or support for redistribution)
- $i_{M}$and $i_{Y}$= regression intercepts
- $e_{M}$and $e_{Y}$= residuals
- $a$= effect of $X$on $M$
- $b$= effect of $M$on $Y$controlling for $X$
- $c^{'}$= direct effect of $X$on $Y$ controlling for $M$

The **indirect effect** is quantified as:

$$ab$$

which represents the portion of the relationship between $X$and $Y$ that is transmitted through $M$.

Because the sampling distribution of the product $ab$is often non-normal, statistical inference relied on nonparametric bootstrapping with 10,000 resamples to generate bias-corrected 95% confidence intervals (Hayes, 2018). An indirect effect is considered present when the confidence interval does not include zero.

# **Supplemental Material F: Manipulation Texts Study 2**

***Deservingness Condition***

Imagine that you live in a small village in a faraway country. People in the village depend on one another. Some people in the village have more money than you, and some have less.

Alex is one of the wealthiest people in the village. However, success has not come easily for Alex. Alex has always been a very hardworking person, and this has made Alex rich. Therefore, you and everyone you know agree that Alex's success is well deserved.

***Ambiguous/Control Condition***

Imagine that you live in a small village in a faraway country. People in the village depend on one another. Some people in the village have more money than you, and some have less.

Alex is one of the wealthiest people in the village.

# **Supplemental Material G: Ruling out Potential Confound (Liking) in Study 2**

To rule out the possibility that the manipulation's effects on, e.g., support for redistribution, are attributable to Alex being perceived as more likable in the deservingness condition, we ran several additional analyses. Since (dis)liking is an essential part of malicious envy, we deemed it not sensible to separate these two (and thus control for liking separately) when testing, e.g., the manipulation's effect on malicious envy. However, it allows us to use malicious envy as a proxy covariate in order to rule out liking as a confound, for example, when testing the effect of the manipulation on support for redistribution and on perceived deservingness.

Results from regression analyses controlling for malicious envy indicated that there was no unintended confounding by likability. More specifically, the effect of the deservingness manipulation (compared to the control condition) on support for redistribution remained significant and similar in size when malicious envy was included as a covariate (*b* = -0.24, *p* = .004, η^2^ = .01) versus without malicious envy as a covariate (*b* = -0.35, *p* < .001, η^2^ = .02).

The same was true when comparing the effects of the manipulation on perceived deservingness, controlling for malicious envy (*b* = 1.49, *p* < .001, η^2^ = .32) versus without malicious envy (*b* = 1.60, *p* < .001, η^2^ = .34).

Finally, re-testing Hypothesis 4 while controlling for malicious envy also produced similar results: Supporting our hypothesis, the IMM was significant (*b* = -0.04, 95% CI [ -0.07, 0.02]), thus indicating that the indirect effect of liberalism on support for redistribution via meritocracy beliefs indeed was moderated by the experimental manipulation (deservingness versus ambiguous/control condition). In further support of Hypothesis 4, results revealed that in the control condition, there was a significant (medium-sized) indirect effect of liberalism on support for redistribution via meritocracy beliefs (*b* = 0.07, 95% CI [ 0.05, 0.11]). The same indirect effect in the deservingness condition was smaller (albeit significant as well; *b* = 0.04, 95% CI [ 0.02, 0.05]). In summary, Hypothesis 4 was also supported when controlling for malicious envy.

# **Supplemental Material H: Results of Study 3 Using the Wealth-Related Envy Subscale**

Since one may argue that the context of wealth is especially relevant for the present manuscript’s theoretical framework, we re-ran all analyses using the 5-item wealth subscale (⍺ = .85) of the Domain-Specific Envy Scale (DSES; Rentzsch & Gross, 2015). Testing Hypotheses 1 and 2, we followed the same procedures as described in Studies 1a and 1b. Again, in support of Hypothesis 1, there was an indirect effect of liberalism on malicious envy (measured with the DSES wealth subscale) via meritocracy beliefs (*b* = 0.007, 95% CI = [0.004, 0.01]; a small-sized effect (Kenny, 2024; Preacher & Kelley, 2011)). Here, the direct effect of liberalism (controlling for meritocracy beliefs) on envy was also significant (*b* = -0.04, *p* = .040).

In order to test Hypothesis 2, we regressed support for redistribution on dispositional wealth-related envy and deservingness beliefs while controlling for household income, education level, age, and gender. Again, there was no significant relationship between dispositional wealth-related envy and support for redistribution (*b* = -0.008, *p* = .768), while meritocracy beliefs did predict support for redistribution (*b* = -0.17, *p* < .001; η^2^ = .03).

# **Supplemental Material I: Testing Interaction Effects with Income**

Given theoretical considerations that malicious envy may be more likely among individuals with fewer economic resources, we conducted additional analyses to examine whether household income moderates (a) the association between liberalism and malicious envy, and (b) the association between malicious envy and support for redistribution. These analyses were not preregistered and are reported here for transparency.

**Liberalism × Income Predicting Malicious Envy**

For each study, we estimated OLS regression models including liberalism, household income, and their interaction. We also computed the simple slope of liberalism at one standard deviation below the mean of income (i.e., among relatively lower-income participants). As shown in Table S2, the interaction term was nonsignificant in all four studies, and the simple slopes at low income were small and nonsignificant. Thus, lower-income liberals were not more likely to report malicious envy than higher-income liberals.

**Malicious Envy × Income Predicting Support for Redistribution**

Next, we tested whether the link between malicious envy and support for redistribution was stronger among lower-income participants. Across Studies 1a, 1b, and 3, neither the interaction nor the simple slope at low income reached significance (Table S3). In Study 2, envy showed a positive association with redistribution among lower-income participants; however, this effect did not replicate across studies. Overall, the results do not support income as a consistent boundary condition for the relationship between malicious envy and redistribution attitudes.

**Interpretation**

Across all studies, income did not systematically moderate (a) the association between liberalism and malicious envy or (b) the association between malicious envy and support for redistribution. Although the idea that lower-income individuals might show stronger envy-related responses is theoretically plausible, the present data provide no consistent evidence for such moderation. Future work with designs specifically tailored to socioeconomic boundary conditions may help clarify when and for whom income shapes envy-related political attitudes.

**Table S2**

*Interaction Effects of Liberalism and Household Income (and Marginal Effects of Liberalism at Household Income 1* SD *Below the Mean) on Malicious Envy.*

| Study | Variable | *b* | *SE* | *t* | *p* | 95% CI |
| --- | --- | --- | --- | --- | --- | --- |
| 1a | Liberalism X income^a^ | -0.05 | 0.05 | -0.89 | .372 | [-0.15, 0.06] |
|  | Liberalism (at income 1 SD below mean) | 0.06 | 0.08 | 0.75 | .455 | [-0.09, 0.21] |
| 1b | Liberalism X income | 0.04 | 0.04 | 1.12 | .265 | [-0.03, 0.11] |
|  | Liberalism (at income 1 SD below mean) | -0.05 | 0.05 | -0.98 | .330 | [-0.15, 0.05] |
| 2 | Liberalism X income | 0.03 | 0.04 | 0.62 | .538 | [-0.06, 0.11] |
|  | Liberalism (at income 1 SD below mean) | 0.03 | 0.06 | 0.55 | .582 | [-0.06, 0.11] |
| 3 | Liberalism X income | -0.008 | 0.02 | -0.42 | .678 | [-0.05, 0.03] |
|  | Liberalism (at income 1 SD below mean) | -0.01 | 0.03 | -0.42 | .673 | [-0.07, 0.04] |
|  |  |  |  |  |  |  |

*Note.* CI = confidence interval; ^a^Measured in 10 categories/steps of $10,000, ranging from 1 (less than $15,000) to 10 (more than $95,000).

**Table S3**

*Interaction Effects of Malicious Envy and Household Income (and Marginal Effects of Malicious Envy at Household Income 1* SD *below the Mean) on Support for Redistribution.*

| Study | Variable | *b* | *SE* | *t* | *p* | 95% CI |
| --- | --- | --- | --- | --- | --- | --- |
| 1a | Malicious envy X income^a^ | -0.007 | 0.05 | -0.15 | .882 | [-0.10, 0.09] |
|  | Malicious envy (at income 1 *SD* below mean) | 0.06 | 0.07 | 0.86 | .390 | [-0.07, 0.18] |
| 1b | Malicious envy X income | 0.02 | 0.03 | 0.67 | .503 | [-0.04, 0.09] |
|  | Malicious envy (at income 1 *SD* below mean) | 0.004 | 0.04 | 0.09 | .926 | [-0.08, 0.09] |
| 2 | Malicious envy X income | 0.04 | 0.04 | 0.93 | .351 | [-0.05, 0.13] |
|  | Malicious envy (at income 1 *SD* below mean) | 0.28 | 0.06 | 4.80 | < .001 | [0.18, 0.40] |
| 3 | Malicious envy X income | 0.02 | 0.02 | 0.89 | .374 | [-0.03, 0.07] |
|  | Malicious envy (at income 1 *SD* below mean) | -0.03 | 0.04 | -0.91 | .363 | [-0.11, 0.04] |

*Note.* CI = confidence interval; ^a^Measured in 10 categories/steps of $10,000, ranging from 1 (less than $15,000) to 10 (more than $95,000).

**References**

Fritz, M. S., & MacKinnon, D. P. (2007). Required sample size to detect the mediated effect. *Psychological Science*, *18*(3), Article 3. https://doi.org/10.1111/j.1467-9280.2007.01882.x

Furnham, A., & Bland, C. (1983). The Protestant work ethic and conservatism. *Personality and Individual Differences*, *4*(2), 205–206. https://doi.org/10.1016/0191-8869(83)90022-3

Harris, C. R., & Henniger, N. E. (2013). Envy, politics, and age. *Frontiers in Psychology*, *4*. https://doi.org/10.3389/fpsyg.2013.00067

Hayes, A. F. (2018). *Introduction to mediation, moderation, and conditional process analysis: A regression-based approach* (Second edition). Guilford Press.

Kenny, D. A. (2024). Mediation (http://davidakenny.net/cm/mediate.htm).

Lin, C.-A., & Bates, T. C. (2021). Who supports redistribution? Replicating and refining effects of compassion, malicious envy, and self-interest. *Evolution and Human Behavior*, *42*(2), 140–147. https://doi.org/10.1016/j.evolhumbehav.2020.08.010

Lin, C.-A., & Bates, T. C. (2024). Support for redistribution is shaped by motives of egalitarian division and coercive redistribution. *Personality and Individual Differences*, *225*, 112684. https://doi.org/10.1016/j.paid.2024.112684

Preacher, K. J., & Kelley, K. (2011). Effect size measures for mediation models: Quantitative strategies for communicating indirect effects. *Psychological Methods*, *16*(2), Article 2. https://doi.org/10.1037/a0022658

Rentzsch, K., & Gross, J. J. (2015). Who turns green with envy? Conceptual and empirical perspectives on dispositional envy. *European Journal of Personality*, *29*(5), 530–547. https://doi.org/10.1002/per.2012

Schoemann, A. M., Boulton, A. J., & Short, S. D. (2017). Determining power and sample size for simple and complex mediation models. *Social Psychological and Personality Science*, *8*(4), Article 4. https://doi.org/10.1177/1948550617715068

Sznycer, D., Lopez Seal, M. F., Sell, A., Lim, J., Porat, R., Shalvi, S., Halperin, E., Cosmides, L., & Tooby, J. (2017). Support for redistribution is shaped by compassion, envy, and self-interest, but not a taste for fairness. *Proceedings of the National Academy of Sciences*, *114*(31), 8420–8425. https://doi.org/10.1073/pnas.1703801114

Van de Ven, N., Hoogland, C. E., Smith, R. H., Van Dijk, W. W., Breugelmans, S. M., & Zeelenberg, M. (2015). When envy leads to schadenfreude. *Cognition and Emotion*, *29*(6), 1007–1025. https://doi.org/10.1080/02699931.2014.961903
